# Secondary Erythrocytosis Among Type 2 Diabetes Mellitus Patients With Hypogonadism Using Sodium‐Glucose Cotransporter 2 Inhibitors and Testosterone Replacement Therapy

**DOI:** 10.1002/edm2.70064

**Published:** 2025-06-03

**Authors:** Maharan Kabha, Hadar Dana, Sameer Kassem, Yoram Dekel, Hilla Cohen, Adnan Zaina

**Affiliations:** ^1^ Department of Urology Lady Davis Carmel Medical Center Haifa Israel; ^2^ Ruth and Bruce Rappaport Faculty of Medicine Technion‐Institute of Technology Haifa Israel; ^3^ Research Authority, Clalit Health Care Organization, Carmel Medical Center Haifa Israel; ^4^ Department of Internal Medicine Carmel Medical Center Palai Kerala India; ^5^ Division of Endocrinology and Metabolism Clalit Medical Health Care Services Haifa Israel; ^6^ The Azrieli Faculty of Medicine Bar‐Ilan University Safed Israel

**Keywords:** diabetes mellitus, erythrocytosis, hypogonadism, SGLT‐2 inhibitors, testosterone replacement therapy

## Abstract

Hypogonadism is commonly linked to type 2 diabetes mellitus (T2DM), with testosterone replacement therapy (TRT) representing a key treatment option. Sodium glucose cotransporter‐2 inhibitors (SGLT‐2i) class is part of T2DM management. Both treatments can increase Hct, Hb and RBC levels with a potential risk for secondary erythrocytosis. This study compares Hct, RBC and Hb changes between T2DM patients treated with and without SGLT‐2i and TRT for hypogonadism.

**Methods:**

Data from Clalit Healthcare Services (2015–2023) was analysed from male T2DM patients with hypogonadism. Mixed linear regression assessed SGLT‐2i effects on Hct, Hb and RBC levels, while generalised estimation equations were used to predict the proportion of patients with Hct > 54%.

**Results:**

In total, 5235 male patients met the inclusion criteria, with 3146 in the SGLT‐2i (+) group, while 2089 comprised the SGLT‐2i (−) group. Mean age was 63.8 ± 11.0 years, mean Hct was 43.3% ± 4.4%, BMI was 30.8 ± 5.2 kg/m^2^ and eGFR was 84.9 ± 19.3 mL/min/1.73m^2^. The SGLT‐2i (+) group demonstrated a statistically significant increase in Hct, Hb, and RBC after TRT initiation (*p* < 0.001). While the overall increase in Hct > 54% was not statistically significant after TRT initiation with OR = 1.85 [95% CI 0.96–3.67], *p* = 0.06. However, in the SGLT2i (+) group, it was significantly higher than for those in the SGLT2i (−) group, OR = 4.85 [95% CI 3.06–7.69], *p* = 0.02.

**Conclusions:**

SGLT‐2i and TRT co‐administration are associated with an increased chance of developing secondary erythrocytosis in T2DM. Awareness and potential treatment discontinuation may prevent unnecessary investigations. Frequent monitoring of these parameters is essential.

## Graphical abstract

Given the frequent association between T2DM and the wide availability and administration of SGLT‐2i as a novel anti‐diabetic class on the one hand and hypogonadism with TRT on the other, concurrent administration may precipitate secondary erythrocytosis. Physician awareness of this potential iatrogenic complication is essential to ensure appropriate monitoring and clinical management.

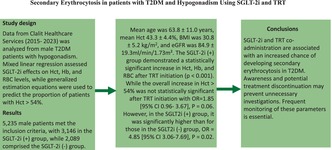

## 1 Background

Diabetes management has been revolutionised recently since the introduction of sodium glucose cotransporter‐2 inhibitors (SGLT‐2i), which have a cardiorenal protective effect beyond glycaemic control [1]. Their use was associated with a modest increase in haematocrit levels, suggesting the cardiovascular positive impact of this crucial anti‐diabetic class by reducing cardiovascular death [2]. The rise in Hct could be one of the mechanisms that play a role in cardiovascular and renal protective effects via improved tissue oxygenation [3]. This effect has been related to increased diuresis secondary to the glycosuria effect of SGLT‐2i, leading to decreased plasma volume and hemoconcentration [2]. Other mechanisms, such as increased erythropoietin (EPO) production by renal fibroblasts, are also likely to play a role [4]. The use of SGLT‐2i has demonstrated an increase in haematocrit by 2%–3% and the associated risk of new‐onset erythrocytosis [5, 6, 7, 8].

Hypogonadism is frequently associated with T2DM, indicating that up to 40% of men with T2DM have testosterone deficiency; however, the etiological link between these two conditions has not been definitively established [9].

It is important to note that testosterone replacement therapy (TRT) represents an essential treatment modality among T2DM patients to restore physiological T levels, particularly when integrated with lifestyle modification and pharmacologic treatment for obesity to improve their health outcomes and quality of life [10]. However, it is well known that TRT might increase up to 3 times the risk of developing secondary erythrocytosis.

It is important to note that, according to current American clinical guidelines, initiation of TRT is contraindicated in patients with baseline erythrocytosis, defined as a haematocrit (Hct) level > 50%. Furthermore, TRT should be discontinued if haematocrit exceeds 54% or if haemoglobin rises above 18.5 g/dL in men due to the potential increased risk of thromboembolic events [8, 11, 12]. This adverse event is dose‐dependent and higher with short‐acting testosterone rather than long‐acting injectable testosterone and transdermal formulations [13]. Furthermore, in randomised control trials, TRT use has resulted in a significant absolute increase of 2%–4% in Hct regardless of treatment duration and testosterone delivery modality [14, 15].

Potential mechanisms that explain the relationship between TRT and Erythrocytosis include the direct stimulation of erythroid progenitor cells and increased kidney production of EPO [12]. Moreover, hepcidin suppression increases iron absorption and transport, enhancing erythrocytosis [16]. Whether TRT increases the risk for thrombotic events remains uncertain [12, 17, 18]. It is worth mentioning that the TRAVERSE study, published recently, demonstrated that TRT was non‐inferior to placebo concerning the incidence of major adverse cardiac events in men with hypogonadism and pre‐existing or at high risk of cardiovascular disease. Nonetheless, recently reported data have shown that TRT increased circulating neutrophils and monocytes and decreased lymphocytes and platelets in men with hypogonadism. Changes in monocyte and neutrophil counts were associated with an increased risk of venous thromboembolism (VTE) [19, 20].

Given the frequent association between T2DM and the wide availability and administration of SGLT‐2i as a novel anti‐diabetic class on the one hand and hypogonadism with TRT on the other hand, the co‐administration of these two classes might cause a further increase in Hct and other haematological parameters.

Therefore, the current study aims to compare haematological parameter changes, including Hct, RBC and Hb, between patients with T2DM treated with SGLT‐2i and TRT for hypogonadism (SGLT2‐i (+) group) and those treated for T2DM with any anti‐diabetic treatment other than SGLT2i with TRT (SGLT‐2i (−) group).

## 2 Materials and Methods

Retrospective data were obtained from Clalit Healthcare Services (CHS) between 2015 and 2023 using an electronic medical records database of male patients aged ≥ 18 years diagnosed with T2DM and hypogonadism according to the International Classification of Diseases (ICD) 9 and 10. Patients were initially divided into two groups according to antidiabetic treatment with SGLT‐2i (+) and without SGLT‐2i (−) before initiation of any testosterone replacement preparation. Notably, treatment with SGLT‐2i included empagliflozin and dapagliflozin. Patients with type 1 DM, Latent Autoimmune Diabetes of Adults (LADA), and advanced chronic kidney disease (CKD) with estimated glomerular filtration rate (eGFR) < 35 were excluded from the study. Additionally, haematological diseases such as polycythaemia vera or haemoglobinopathies such as thalassaemia and haematocrit > 50% were excluded from the study.

### 2.1 Statistical Analysis

We used mixed linear regression analyses to determine the effect of SGLT‐2i treatment on Hct, Hb and RBC levels, as indicated in laboratory results. Each blood test level was predicted by group, time pre‐treatment, and post‐treatment with TRT and their interaction. The haematological blood test date was selected up to 6 months before treatment initiation with TRT in both groups with and without SGLT‐2i. The post‐treatment blood test date was selected to be at least 3 months after the start of TRT treatment and up to 1 year after TRT initiation.

To determine the effect of treatment modality concerning the proportion of patients who have Hct > 54%, we used generalised estimation equation (GEE) regression analyses, where the proportion of Hct > 54% was predicted by group SGLT‐2i (−) group vs. SGLT‐2i (+) group, time pre‐treatment and post‐treatment with TRT, and the interaction between them. This Hct cut‐off was selected in alignment with current clinical guidelines, which recommend discontinuing TRT in patients who reach this level.

All regression analyses were controlled for pre‐treatment eGFR levels. Simple mean analysis was used to quantify the interaction effect. The R Foundation for Statistical Computing version 4.3.1 was used to perform the analysis. The Carmel Hospital Institutional Review Board (Helsinki Committee) approved the study.

## 3 Results

We identified 5235 male patients with T2DM and hypogonadism who met our inclusion criteria. Of these, 3146 participants were in the SGLT‐2i (+) group, while 2089 comprised the SGLT‐2i (−) group. The mean age was 63.8 ± 11.0 years, mean Hct was 43.3% ± 4.4%, BMI was 30.8 ± 5.2 kg/m^2^, and eGFR was 84.9 ± 19.3 mL/min/1.73 m^2^. Other baseline characteristics are summarised in Table 1.

**TABLE 1:** Baseline characteristics of patients with diabetes and hypogonadism at TRT initiation.

Variable	Total (*n* = 5235)	SGLT‐2i (−) (*n* = 2089)	SGLT‐2i (+) (*n* = 3146)	*p*
Age, mean ± SD	63.8 ± 11	61.9 ± 12.4	65.1 ± 9.8	< 0.001
BMI, kg/m^2^, mean (SD)	30.8 (5.2)	30.7 (5.6)	30.9 (5)	0.31
Hb, g/dL, mean (SD)	14.2 (1.5)	14.2 (1.5)	14.2 (1.5)	0.25
RBC, mil/μL, mean (SD)	5 (0.6)	4.9 (0.6)	5 (0.6)	< 0.001
Hct, %, mean (SD)	43.3 (4.4)	43.1 (4.4)	43.5 (4.5)	< 0.001
eGFR, mL/min/1.73 m^2^, mean (SD)	84.9 (19.3)	87.5 (19.1)	83.2 (19.3)	< 0.001
Current smoker, *n* (%)	909 (19.3)	376 (20.0)	533 (18.8)	0.30

Abbreviations: BMI, body mass index; eGFR, estimated glomerular filtration rate; Hb, haemoglobin; Hct, haematocrit; RBC, red blood count; TRT, Testosterone replacement therapy.

**FIGURE 1:**
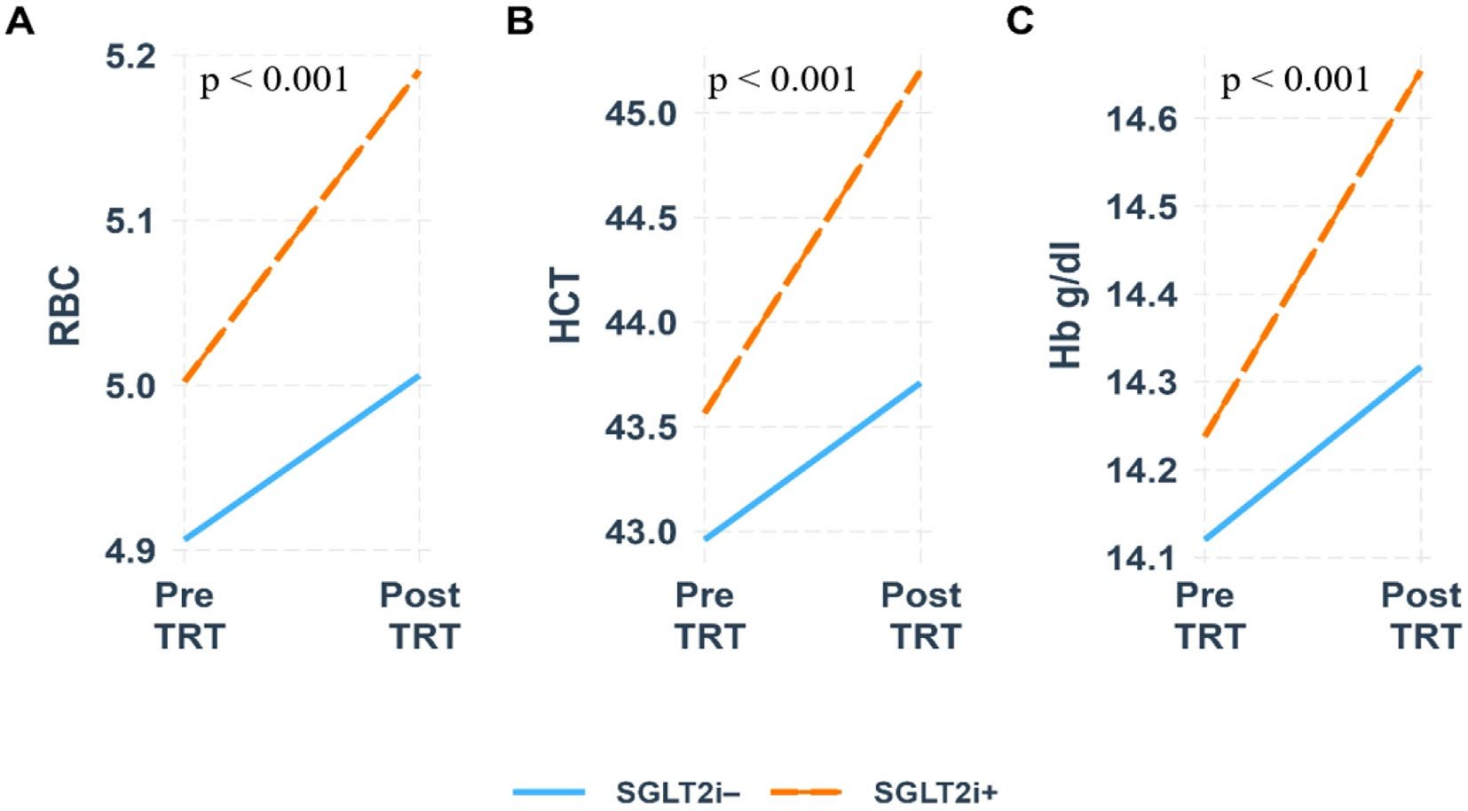
Illustrates the RBC, Hct and Hb levels in both groups before and after TRT treatment. Hb, haemoglobin; Hct, haematocrit; RBC, red blood count; SGLT‐2i, sodium‐glucose cotransporter‐2 inhibitors; TRT, testosterone replacement therapy.

For all three models, the interaction of time and group is significant, indicating that the dependent variable changes differently across time and group. In the SGLT‐2i (+) group, the measured haematological parameters significantly increased after TRT treatment in all models. For RBC, there was a significant increase in individuals treated with SGLT‐2i (+) compared to SGLT‐2i (−) [*B* = 0.19 vs. 0.10, *p* < 0.001]. Hct also showed a marked increase in the SGLT‐2i (+) group compared to the SGLT‐2i (−) group [*B* = 1.64 vs. 0.75, *p* < 0.001]. Similarly, Hb levels demonstrated a significant increase in the SGLT‐2i (+) group compared to SGLT‐2i (−) [*B* = 0.41 vs. 0.19, *p* < 0.001] (Figure 1).

**FIGURE 2:**
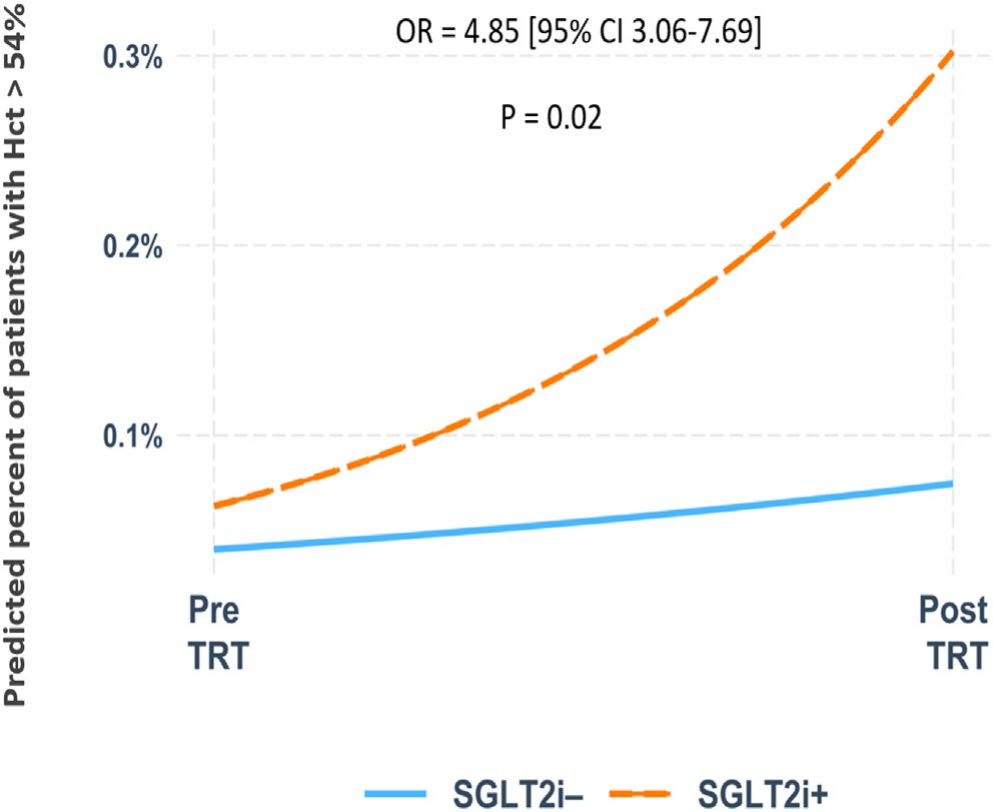
The predicted proportion of Hct > 54% after treatment with TRT between the two groups.

We identified 28 (1.3%) patients with Hct > 54% in the SGLT‐2i (−) group after initiating TRT, while in the SGLT‐2i (+) group, 113 (3.6%) patients were identified with Hct > 54%. The generalised estimation equation (GEE) regression analyses to determine the effect of SGLT‐2i treatment concerning the proportion of patients who have Hct > 54% revealed that overall, the proportion of those with Hct > 54% increases after treatment with TRT, but it is not statistically significant OR = 1.85 [95% CI 0.96–3.67], *p* = 0.06. However, for patients in the SGLT‐2i (+) group, the likelihood of an increase in Hct > 54% is significantly higher than for those in the SGLT‐2i (−) group OR = 4.85 [95% CI 3.06–7.69] vs. 1.88 [95% CI 0.96–3.63], *p* = 0.02. (Figure 2).

## 4 Discussion

The current research results disclosed new data regarding the effects of TRT among patients with hypogonadism and T2DM under treatment with SGLT‐2i compared to other anti‐diabetic agents. Notably, at baseline and before initiating TRT as expected, we found a significant absolute increase in Hct, RBC, and Hb among patients using SGLT‐2i. These results, as expected, demonstrated the already known effects of SGLT‐2i. The post/pre‐treatment changes with TRT in the above parameters were more pronounced in the group using SGLT‐2i. These changes were demonstrated by analysing the effect of time on these parameters, emphasising the need for more frequent measurements of these haematological parameters, particularly during the first year of initiating combination treatment. Notably, the increased risk of erythrocytosis was reported among patients using SGLT‐2i and TRT separately. However, reviewing the literature, scant data were published on patients using combination treatment. Recently, Gosmanov AR et al. reported a comprehensive analysis of an extensive healthcare database suggesting that combined treatment of SGLT‐2i and TRT may display a higher relative risk of new‐onset erythrocytosis. However, the absolute risk remained low [21].

Suppressed hepcidin hormone, produced mainly by the liver, represents a potential mechanism that stimulates erythropoiesis and might induce erythrocytosis. Notably, hepcidin, the master iron regulatory peptide, performs its function via the hepcidin‐ferroportin interaction [22]. Suppressing hepcidin entails excessive intestinal absorption of iron and increased iron release by macrophages. Moreover, macrophages and enterocytes exhibit strongly upregulated ferroportin expression in the erythropoietic response in iron‐restricted conditions [23]. Increased EPO production represents another essential factor demonstrated among patients using TRT and T2DM using SGLT‐2i. It is noteworthy that Aoun et al. recently addressed the pathophysiology of erythrocytosis and the hypothetical link between SGLT‐2i use and activation of hypoxia‐inducible factor 2α (HIF‐2α) in some cases with unmasked PV [24, 25]. The presence of the hemochromatosis gene mutation without clinical manifestations until SGLT‐2i and TRT were prescribed was reported.

Studies demonstrated the cardiovascular safety of TRT and the cardiorenal protective effect of SGLT‐2i. Nonetheless, it is crucial to be vigilant about the appearance of acquired erythrocytosis in such a combination and to consider treatment discontinuation when needed before starting further unnecessary investigation to identify other aetiologies that cause erythrocytosis, such as polycythemia vera (PV) or underlying benign or malignant neoplasms [26, 27, 28].

The strength of our current study is that the results are based on the analyses of patients' records from an extensive electronic registry database of Clalit Health Services over a 9‐year period. This provides a solid representation of a population at risk for erythrocytosis associated with concomitant administration of SGLT2‐i and TRT in hypogonadal patients with T2DM.

The following limitations of our study should be acknowledged: First, this is a retrospective study. Second, all available androgen replacement therapies, short‐acting and long‐acting injectable derivatives, and transdermal formulations were designed as TRT treatments; third, the study did not specifically evaluate patients with obstructive pulmonary disease as a potential confounding factor that might influence haematological parameters. Nonetheless, other essential factors such as smoking, eGFR, and BMI that might influence haematological parameters were incorporated into the analysis.

## 5 Conclusions

The current study demonstrated a significant increase in the risk of secondary erythrocytosis following the initiation of TRT in patients with T2DM already treated with SGLT‐2i. Increasing the vigilance of this possible complication among health care practitioners and treatment discontinuation might avoid unnecessary investigation. Therefore, monitoring these haematological parameters at least quarterly during the first year of TRT is strongly recommended to mitigate potential complications and ensure patient safety. Further studies are needed in this field.

## Author Contributions

Maharan Kabha wrote the study protocol and reviewed the results. Hadar Dana performed the statistical analysis and prepared the table. Sameer Kassem reviewed the results and the manuscript. Yoram Dekel reviewed the results and the manuscript. Hilla Cohen extracted the database records and drew the figures. Adnan Zaina prepared the study protocol and wrote the manuscript. All authors revised and approved the final manuscript.

## Conflicts of Interest

The authors declare no conflicts of interest.

## Data Availability

The data supporting this study's findings are available from the corresponding author upon reasonable request.
